# Treatment for Recurrent Differentiated Thyroid Cancer: A Canadian Population Based Experience

**DOI:** 10.7759/cureus.7122

**Published:** 2020-02-27

**Authors:** Aria Shokoohi, Eric Berthelet, Sabrina Gill, Eitan Prisman, George Sexsmith, Eric Tran, Adam White, Sam M Wiseman, Jonn Wu, Cheryl Ho

**Affiliations:** 1 Medical Oncology, BC Cancer, Vancouver, CAN; 2 Radiation Oncology, BC Cancer, Vancouver Cancer Centre, Vancouver, CAN; 3 Endocrinology, St. Paul's Hospital, Vancouver, CAN; 4 Otolaryngology, Vancouver General Hospital, Vancouver, CAN; 5 Nuclear Medicine, St. Paul's Hospital, Vancouver, CAN; 6 Surgery, St. Paul’s Hospital & University of British Columbia, Vancouver, CAN; 7 Medicine, University of British Columbia, Vancouver, CAN

**Keywords:** differentiated thyroid cancer, recurrence, salvage, radioactive iodine, radiotherapy

## Abstract

Introduction: Management of recurrent differentiated thyroid cancer (DTC) may include surgery, radioactive iodine (RAI), and external beam radiotherapy (EBRT). Systemic therapy may also be offered for RAI-refractory DTC. The study objective was to review patterns of practice in British Columbia (BC) for treatment of recurrent DTC, assess rates of RAI-refractory disease, and evaluate outcomes.

Methods: BC Cancer provides cancer care to a population of 4.6 million. A retrospective review of all patients with DTC stage I-IVB disease referred to BC Cancer from 2009 to 2013 was conducted. Patient and DTC characteristics, locoregional and distant recurrence, surgical management, RAI, EBRT, and systemic therapy details were retrospectively collected. Relapse-free survival (RFS), overall survival (OS), and disease-specific survival (DSS) were calculated using the Kaplan-Meier method.

Results/Discussion: Some 1062 DTC patients were identified. Median follow-up was 4.1 years. Baseline characteristics: female 74%, median age 50, papillary/follicular/Hurthle cell 92%/6%/2%. Stage at presentation: I 60%, II 8%, III 22%, IVA/IVB 10%. Locoregional and/or distant recurrence occurred in 136 patients (13%). Locoregional recurrence (n=118) was treated with surgery +/- RAI or EBRT 48%, RAI +/- EBRT 40%, EBRT alone 1%, 11% were observed without treatment. Some 27 patients had a second cancer recurrence. Some 37 patients (3%) developed distant metastatic disease and common sites of distant metastases were: lung 76%, bone 30%, and liver 8%. Some 27 cases (2%) were deemed RAI-refractory. Some six patients (0.6%) received systemic therapy with a vascular endothelial growth factor tyrosine kinase inhibitor (VEGF TKI). Five-year RFS was calculated to be 82%, OS 95%, and DSS 98% for the study population.

Conclusions: In our population-based study cohort, 87% of patients were rendered disease-free by primary disease management. Multi-modality treatment of locoregional recurrence facilitated disease-free status in the majority of patients (67%). RAI-refractory disease developed in 2% of patients and despite a significant number of metastatic recurrences, only a small number of patients received systemic therapy.

## Introduction

Differentiated thyroid cancer (DTC) comprises the vast majority of thyroid cancers. In general, patients diagnosed with DTC have an excellent prognosis, with a 10-year overall survival (OS) of greater than 90%. The guidelines for initial management, as set by the American Thyroid Association (ATA), for DTC patients aim to improve overall and disease-specific survival (DSS) and reduce the risk of persistent/recurrent disease and associated morbidity [1]. This is achieved by removing the primary tumor and any metastatic lymph nodes surgically, and by providing adjuvant radioactive iodine (RAI), external beam radiation (EBRT), and/or thyroid stimulating hormone (TSH) suppression [2].

Most DTC patients have an excellent prognosis after their primary treatment. However, disease recurrence may affect up to 30% of patients within 10 years of their initial diagnosis, depending upon the cancer stage and treatment [3]. The most common pattern of failure for DTC patients is regional or cervical nodal recurrence, which is associated with an increased mortality rate. Both serum thyroglobulin (TG) measurement and RAI scanning have a high yield in detecting disease recurrence with a combined sensitivity of greater than 90% and are therefore used routinely for the post-treatment monitoring of these patients [4]. The guidelines recommend monitoring through clinical exam, TG measurement, and/or imaging every 6-24 months depending on the patient’s initial cancer risk stratification, initial therapy, and response to therapy [1]. 

Most patients diagnosed with recurrent DTC undergo salvage treatment with further surgery and/or RAI therapy [5-6]. A small proportion of patients have locoregional disease that is not amenable to local treatment or have metastases refractory to RAI ablation. These patients are potentially eligible for palliative external beam or stereotactic body radiotherapy or systemic therapy with tyrosine kinase inhibitors like sorafenib or lenvatinib [7-8].

The study objective was to review the multidisciplinary treatment and outcomes of DTC patients in order to determine the natural history of their disease and the impact of different treatment modalities including surgery, RAI, EBRT, and systemic therapies on patients’ outcomes. With the introduction of systemic therapy into the treatment algorithm, the secondary study objective was to determine the proportion of patients who develop RAI-refractory (RAI-R) disease through the course of their treatment.

## Materials and methods

A retrospective review of all patients referred to BC Cancer between January 1, 2009 and December 31, 2013 for management of pathologically confirmed DTC stage I-IVB disease was performed. Patients who did not undergo thyroid surgery or had metastatic disease at presentation were excluded from the study population.

Patient and tumor characteristics were extracted from the Outcomes and Surveillance Integrated System (OaSIS) and by chart review. Data collected included: age at diagnosis, sex, histology, and cancer stage. The AJCC 7th edition TNM system was used for staging. Initial and subsequent management including type of surgery, RAI utilization, radiotherapy dose, fractionation, and location of radiation and use of systemic therapy were collected.

Patients were considered to have recurrence with the reappearance of the disease at least six months after the date they completed their primary management and any of the following occurred: cytologically or histologically proven lymph nodes or locoregional tumor recurrence, iodine-131 whole-body scan or other imaging modality consistent with locoregional or metastatic disease, and biochemical evidence of a persistently elevated level of TG. The location (locoregional or distant) and treatment of the recurrent disease were also recorded.

Locoregional recurrence was defined as cancer being located in the central or lateral neck and distant recurrence as cancer being located in distant sites. RAI-R disease was defined according to at least one of the following criteria: at least one lesion without iodine uptake on any RAI scan, progression on radiographic imaging within 12 months after RAI therapy despite iodine-131 avidity at the time of initial treatment, or cumulative delivery of RAI that was clinically deemed to have reached the maximum dose.

Statistics were carried out using SPSS software version 25 (SPSS Inc, Chicago, IL) and variables were compared using the t-test, Chi-square, or Mann-Whitney tests. The disease status at last follow-up, date of death, and cause of death were also recorded. Relapse-free survival (RFS) was defined as date of diagnosis to the date of biochemical, radiographic, or pathologic evidence of recurrence or death due to DTC with living patients censored on date of their last follow-up or unrelated death. OS was defined as being from the date of diagnosis to the date of death from any cause, with living subjects censored at last follow-up. Disease specific survival (DSS) was defined as being from the date of diagnosis to the date of death from DTC, with living subjects censored at their last follow-up or unrelated death. RFS, OS, and DSS were calculated using the Kaplan-Meier method and compared with the log rank test. 

The study was approved by the BC Cancer research ethics board.

## Results

Some 1062 patients with pathologically confirmed DTC (stages I-IVB) were referred to BC Cancer during the study period representing 65% of all cases diagnosed in BC. Baseline characteristics are summarized in Table 1. There were 782 female (74%) patients in the study population. The median age at diagnosis was 50 years (range 9-92 years). Some 976 (92%) patients presented with papillary carcinoma (PTC), 59 (6%) with follicular carcinoma (FTC), and 27 (2%) with Hurthle cell carcinoma (HCC). Cancer stage at presentation using the AJCC 7th edition was: I 60%, II 8%, III 22%, and IVA/IVB 10%.

**Table 1:** Patient and tumor characteristics of study differentiated thyroid cancer patient population (recurrent vs non-recurrent).

Patient and tumor characteristics	Non-recurrent (n = 926)	Recurrent (n = 136)	P-value
Age at diagnosis, median (interquartile range), years	49 (39-59)	53 (37-67)	0.045
Age at diagnosis, ≥ 45 years, no (%)	580 (63%)	90 (66%)	0.424
Sex, F	701 (76%)	81 (60%)	<0.001
Follow-up, median (interquartile range), years	3.9 (2.3-5.3)	5.1 (3.9-6.5)	<0.001
Histology – no. (%)			0.328
Papillary	853 (92%)	123 (91%)	
Follicular	52 (6%)	7 (5%)	
Hurthle cell	21 (2%)	6 (4%)	
AJCC 7^th^ Edition Pathological Stage – no. (%)			<0.001
Stage I	585 (63%)	52 (38%)	
Stage II	75 (8%)	7 (5%)	
Stage III	186 (20%)	44 (33%)	
Stage IVA	79 (9%)	33 (24%)	
Stage IVB	1 (<1%)	0 (0%)	
Pathology – no. (%)			
Multifocal disease	423 (46%)	81 (60%)	<0.001
Extrathyroidal extension	238 (26%)	69 (54%)	<0.001
Vascular invasion	113 (12%)	42 (31%)	<0.001
Size of tumor, mean (range), cm	2.0 (0.02-13.0)	3.3 (0.15-9.0)	<0.001
Resection margins, positive	227 (26%)	72 (56%)	<0.001

The initial management of the patients is summarized in Table 2. Surgical intervention included 66 (6%) lobectomies and 996 (94%) total/near-total thyroidectomies. Nodal management of disease included 141 (13%) central neck dissection only, 108 (10%) selective lateral neck dissection only, and 49 (5%) both central and lateral neck dissections. Lateral neck dissections were therapeutic, while central neck dissections may have been prophylactic or therapeutic. For adjuvant treatment, RAI was administered to 692 patients (65%) and radical EBRT was given to 51 patients (5%).

**Table 2:** Initial management, recurrence, and outcome of differentiated thyroid cancer patients (recurrent vs non-recurrent). RAI, radioactive iodine

	Non-recurrent (n = 926)	Recurrent (n = 136)	P-value
Surgical Intervention			0.038
Lobectomy	63 (7%)	3 (2%)	
Total/near-total thyroidectomy	863 (93%)	133 (98%)	
Nodal Management			<0.001
Central neck dissection only	118 (13%)	23 (17%)	
Lateral neck dissection only	80 (8%)	28 (21%)	
Central and lateral neck dissections	36 (4%)	13 (9%)	
None	692 (75%)	72 (53%)	
RadioIodine-131 Treatment			<0.001
Yes	559 (60%)	133 (98%)	
Thyrogen use	445 (80%)	106 (80%)	
RAI dose, median (interquartile range), mCi	75 (48-100)	99 (50-116)	
No	330 (36%)	3 (2%)	
Unknown	37 (4%)	0 (0%)	
External Beam Radiation			<0.001
Adjuvant	37 (4%)	14 (10%)	
None	889 (96%)	122 (90%)	
Time to first recurrence, mean (range), months	N/A	29.4 (7.9-70.4)	
Time to locoregional recurrence, mean (range), months (n = 118)	N/A	29.9 (7.9-70.4)	
Time to metastatic recurrence, mean (range), months (n = 37)	N/A	38.3 (7.9-87.3)	
Method of Detection – First Recurrence			
Imaging	N/A	61 (45%)	
Pathologic confirmation	N/A	49 (36%)	
Thyroglobulin	N/A	26 (19%)	
Location of Recurrence			
Locoregional	N/A	99 (73%)	
Locoregional → Distant	N/A	19 (14%)	
Distant	N/A	18 (13%)	
Outcome			<0.001
Alive, no disease	897 (97%)	78 (57%)	
Alive, with disease	0 (0%)	39 (29%)	
Dead, from disease	0 (0%)	17 (13%)	
Dead, unrelated causes	29 (3%)	2 (1%)	

**Figure 1:**
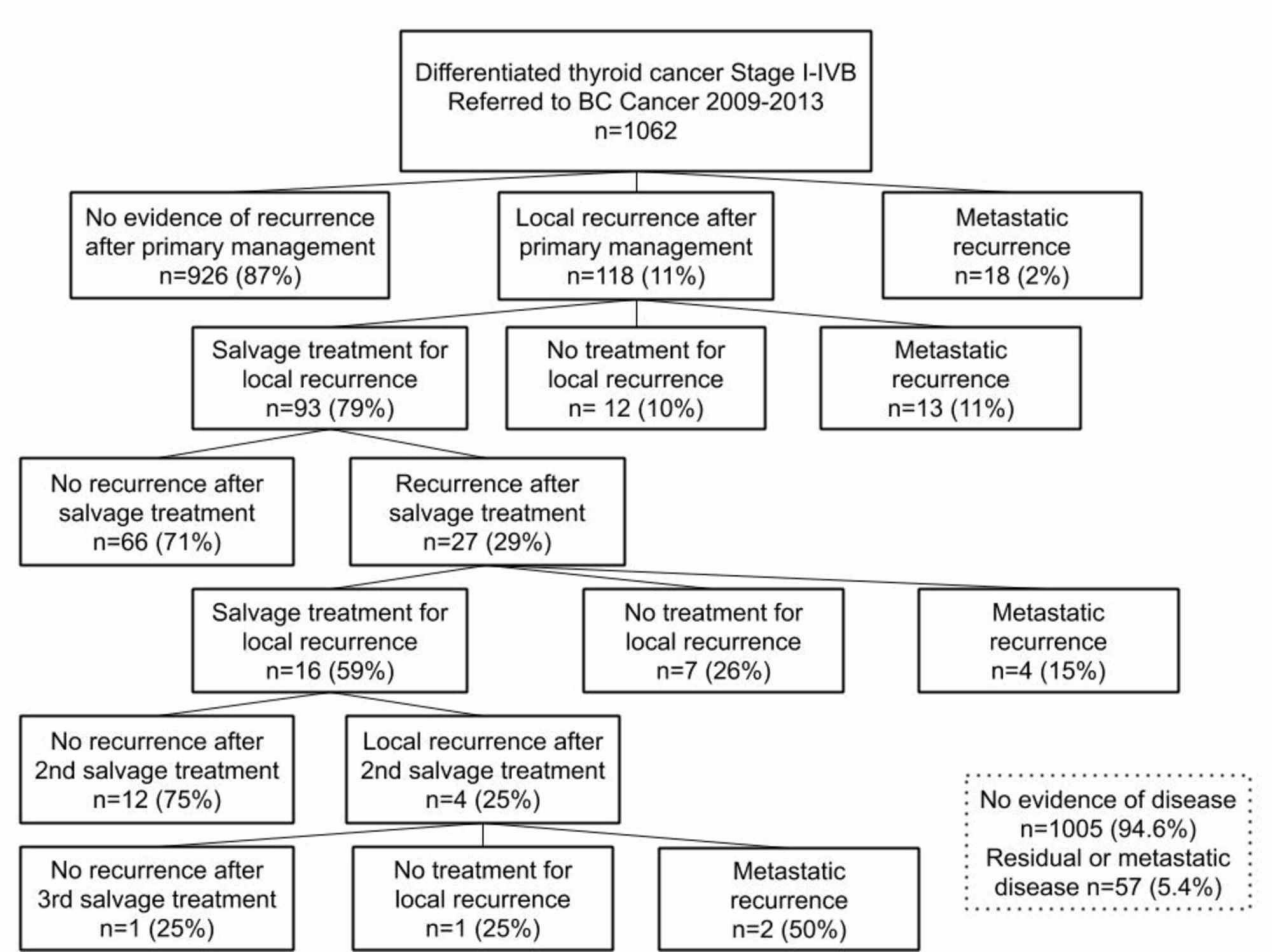
DTC stage I-IVB from 2009 to 2013 patient flow (n=1062). DTC, differentiated thyroid cancer

**Figure 2:**
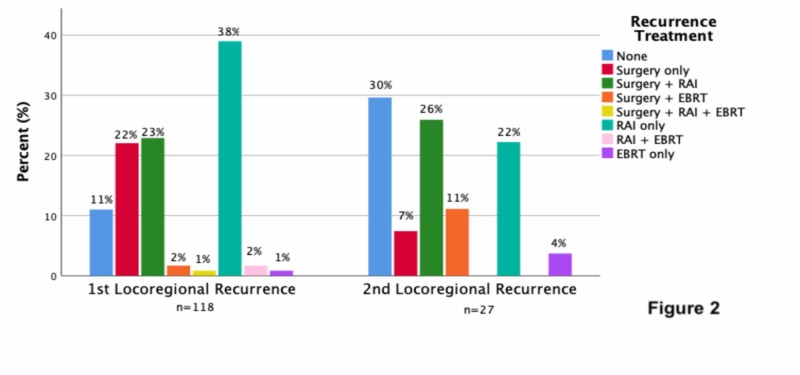
Management of first locoregional recurrence DTC (n = 118) and second locoregional recurrence DTC (n = 27). DTC, differentiated thyroid cancer; RAI, radioactive iodine; EBRT, external beam radiotherapy

No evidence of recurrence was observed in 926 (87%) patients following their primary management (Figure 1). Locoregional and/or distant recurrence occurred in 136 patients (13%). The multi-modality treatment of patients with locoregional recurrence is summarized in Figure 2. Some 118 of the study population (11%) with locoregional recurrence were treated with surgery +/- RAI or EBRT 48%, RAI +/- EBRT 40%, EBRT alone 1%, and 11% were observed without treatment. Some 27 (3%) patients experienced a second locoregional recurrence and were treated with surgery +/- RAI or EBRT 44%, RAI +/- EBRT 22%, EBRT alone 4%, and 30% were observed without treatment. Locoregional recurrence was successfully managed in 79 patients (67%) with no evidence of residual disease. Some 8 of the 20 patients (40%) who did not receive further treatment following their locoregional recurrence were found to have RAI-R disease.

**Figure 3:**
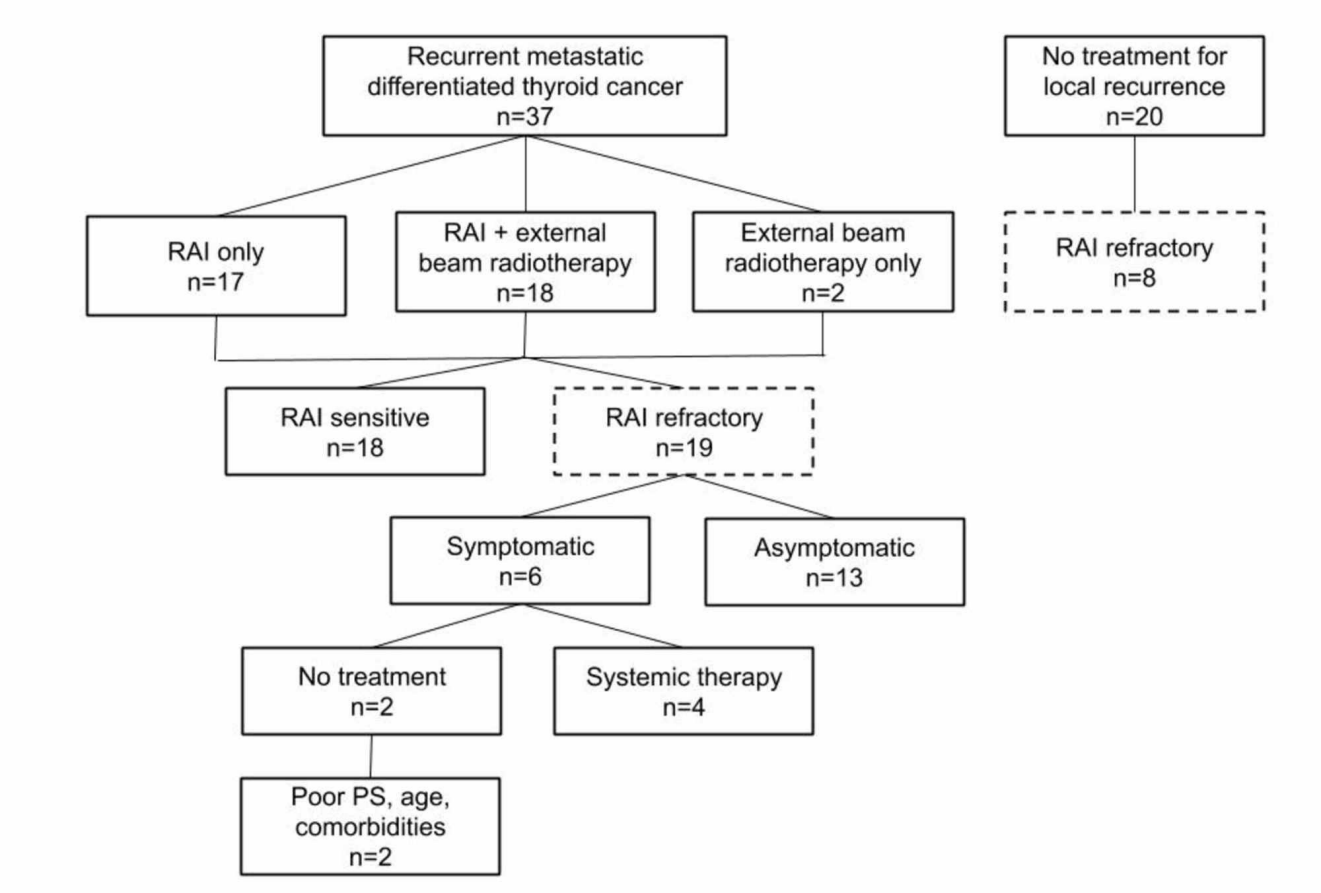
Recurrent metastatic DTC treatment patient flow. DTC, differentiated thyroid cancer; RAI, radioactive iodine; PS, performance status

Combining the patients with locoregional recurrence who did not receive any treatment (n=20) and those with metastatic disease (n=37), 27 of the 57 patients (47%) developed RAI-R disease (2% of the study population) (Figure 3). RAI-R disease was diagnosed due to a lack of RAI uptake in 24 patients (89%) and maximum RAI dose in three patients (11%). Out of the 37 patients (3% of the study population) who developed distant metastatic disease, 19 (51%) had a prior locoregional recurrence. Common sites of distant metastases were lung (76%), bone (30%), and liver (8%). Figure 3 summarized the treatment of the 37 patients who developed distant metastatic disease: RAI treated alone 46%, RAI + EBRT treated 49%, and EBRT treated alone 5%. Some 19 patients with metastatic disease were diagnosed with RAI-R disease. Out of the six symptomatic patients, four (67%) were treated with a multi-kinase inhibitor. The remaining two patients were not treated due to older age, poor performance status (PS), and underlying comorbidities. Two patients who did not demonstrate RAI uptake at diagnosis received systemic therapy. Of the entire study cohort, six patients (0.6%) received systemic therapy with a vascular endothelial growth factor tyrosine kinase inhibitor (VEGF TKI).

**Figure 4:**
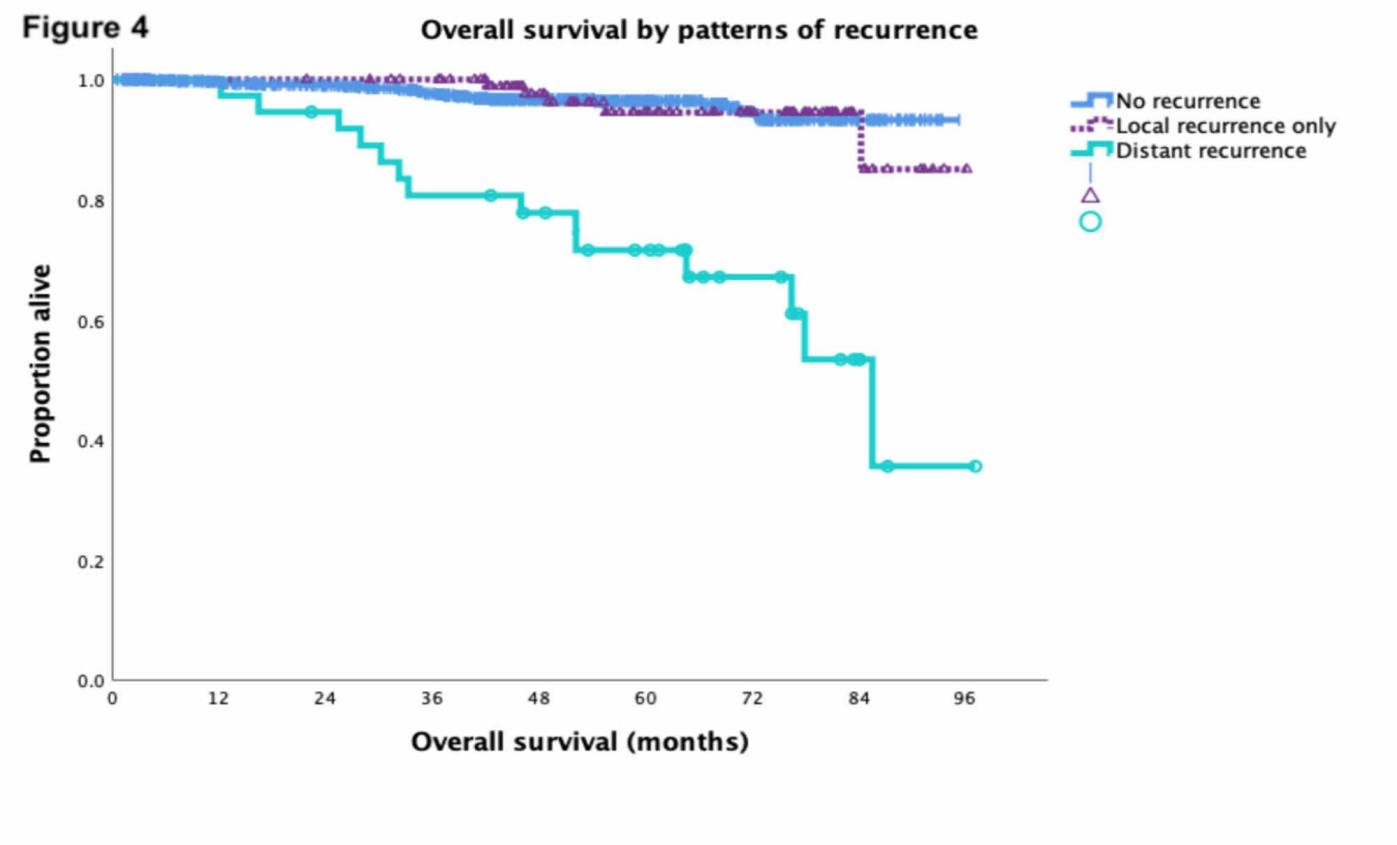
Year OS for no recurrence 96% (n = 926), locoregional recurrence 95% (n = 99), and distant recurrence 72% (n = 37) (p < 0.001). OS, overall survival

Median study patient follow-up was 4.1 years. There were 975 patients (92%) who had no evidence of disease, 39 patients (4%) who were alive with disease, 17 patients (1%) who died of disease, and 31 patients (3%) who died of other causes (Table 1). The five-year RFS, OS, and DSS were 82%, 95%, and 98% respectively for all patients. OS for locoregional and distant recurrence is shown in Figure 4. Five-year OS for those with no recurrence was 96%, those with locoregional recurrence was 95%, and those with distant recurrence was 72% (p < 0.001).

## Discussion

The current study outlines the natural history of a contemporary cohort of DTC patients from initial management to salvage treatment of recurrent disease and the development of RAI-R disease. Overall, 87% were cured with primary management and 67% of the locally recurrent patients were cured of DTC with multimodality therapy. Of all the study patients, only 2% developed RAI-refractory disease and only a small number received systemic therapy.

The incidence of DTC has been steadily rising. A SEER database study in 2017 noted an increase in the incidence and mortality of thyroid cancer between 1994 and 2013 that was 3.6% and 1.1% annually [9]. While in part this trend has been attributed to overdiagnosis, the rising mortality rate suggests that there are other factors that must be considered. Exposure to ionizing radiation at a young age has a well-established causative role for DTC development and other factors including obesity and exposure to endocrine-disrupting chemicals may also have an impact on the incidence of disease [10-12]. These potential etiological factors may also contribute to the diagnosis of more aggressive or higher stage DTC.

With rising incidence of DTC, the burden on the healthcare system becomes increasingly relevant. From examining the degree of surgical intervention needed for low risk incidentally found disease to managing the more aggressive variants, the continuum of DTC treatment has increasing healthcare resource demands. To date there are no contemporary longitudinal studies from patient diagnosis to the development of metastatic disease that provide a current perspective regarding the prevalence of this disease. Our study suggests that in the current era of DTC management only 13% of patients recur after primary therapy, 3% go on to develop metastatic disease, and 2% eventually become RAI-R. It is this small subset of DTC patients that may be considered candidates for systemic therapy.

The ATA guidelines recommend multimodal salvage treatment for recurrent DTC [1]. The role of surgical intervention to optimally manage recurrent gross disease must be balanced against risks of surgery. Prior studies have evaluated the management of recurrent nodal disease. Wang et al. observed a 3% locoregional recurrence rate for PTC in a cohort of 3664 patients [5]. Curative intent salvage therapy rendered 80% free of disease as 12% developed recurrent locoregional disease and 8% distant metastatic recurrence. A study from MD Anderson of patients with recurrent or persistent DTC who were treated with comprehensive lateral neck dissection of level II-V demonstrated an infield lateral neck control rate of 96% and disease specific survival of 91% at 10 years [6]. Our data for salvage DTC treatment is consistent with the literature although the proportions may be somewhat lower due to the inclusion of both locoregional and distant recurrence. All studies consistently support aggressive multimodality management for locally recurrent DTC.

Our understanding of the evolution of metastatic disease is informed by a retrospective case series reported by Durante et al. who examined a cohort of 444 metastatic DTC patients treated between 1953 and 1994 in order to assess the benefits and limitations of RAI [13]. Effective salvage with RAI was achieved in 43% of the 295 patients that exhibited RAI uptake with no subsequent evidence of residual disease. However, of the 444 patients, 168 (38%) received RAI but did not achieve cure and 132 (30%) did not demonstrate RAI uptake, suggesting that RAI-R disease develops in 68% of patients with metastatic disease. The RAI uptake without cure patients and no RAI uptake patient cohorts constitute a portion of the modern-day definition of RAI-R. The cumulative dose of RAI was up to 1495 mCi and therefore there likely were additional patients that would now be considered RAI-R. The high rates of RAI-R disease in this 1950s-1990s era may be a function of diagnosis at later stages and less aggressive surgical salvage options.

The DECISION and SELECT trials demonstrated that sorafenib and lenvatinib, respectively, achieved a progression-free survival (PFS) benefit over placebo in patients with RAI-R DTC [7-8]. The definition of RAI-R in the trials included the presence of at least one target lesion without RAI uptake, tumor that had RAI uptake and progressed within 12-16 months of treatment, tumor that progressed after two RAI treatments within 16 months of each other, or received a cumulative RAI dose of > 600 mCi. Durante et al.’s study suggests that the majority of patients with metastatic DTC ultimately become RAI-R although it does not identify those individuals who are asymptomatic and do not require systemic therapy. In our study, of the patients harboring metastatic disease, 68% were asymptomatic and did not require further therapy during the study period. There were 17 patients (2%) who died of thyroid cancer of which 11 (65%) did not receive systemic therapy despite availability; acknowledging limitations due to patient factors, this patient subset could potentially represent individuals who missed an opportunity for systemic treatment.

Radiotherapy techniques for oligometastatic disease may also be employed. A study by Hamilton et al. explored the use of stereotactic body radiotherapy (SBRT) and noted excellent rates of local control (88% at two years and 79% at three years) and relatively low morbidity [14]. That study suggested that there will likely be an increase in the use of SBRT for DTC patients with oligometastases that have a discordant response to RAI.

Our study has several limitations that must be reviewed. Based on provincial referral patterns only 65% of DTC cases in British Columbia (BC) were included in our study population presumably because low risk disease may not be referred to BC Cancer. DTC has a long natural history and our median duration of follow up was limited to 4.1 years. In addition, because of the study era, the rate of total thyroidectomy versus lobectomy was higher than it would be currently due to changes in national guidelines that have impacted surgical practice, this may influence recurrence rates estimates. Our study strengths include our ability to collect detailed information during the course of each patient’s disease and the availability of complete records of radiotherapy and systemic treatment details due to a single payer Canadian healthcare system.

## Conclusions

In our population-based DTC cohort, 87% of patients were cured by primary disease management. Locoregional DTC recurrence was successfully managed with surgery, RAI and/or EBRT with no evidence of residual disease in two-thirds of patients. Multi-modality treatment of locoregional recurrence facilitates complete disease ablation in the majority of patients. However, in our study, 17 patients died of DTC and only six received systemic therapy. Our longitudinal study suggests that aggressive salvage management is warranted for recurrent DTC and surveillance strategies should ensure that all patients appropriate for salvage oligometastatic SBRT and TKI therapy receive treatment.

## References

[1] Haugen BR, Alexander EK, Bible KC (2016). 2015 American Thyroid Association Management Guidelines for Adult Patients with Thyroid Nodules and Differentiated Thyroid Cancer: The American Thyroid Association Guidelines Task Force on Thyroid Nodules and Differentiated Thyroid Cancer. Thyroid.

[2] Mazzaferri EL (2000). Long-term outcome of patients with differentiated thyroid carcinoma: effect of therapy. Endocr Pract.

[3] Mazzaferri EL, Jhiang SM (1994). Long-term impact of initial surgical and medical therapy on papillary and follicular thyroid cancer. Am J Med.

[4] Webb RC, Howard RS, Stojadinovic A, Gaitonde DY, Wallace MK, Ahmed J, Burch HB (2012). The utility of serum thyroglobulin measurement at the time of remnant ablation for predicting disease-free status in patients with differentiated thyroid cancer: a meta-analysis involving 3947 patients. J Clin Endocrinol Metab.

[5] Wang LY, Migliacci JC, Tuttle RM, Shaha AR, Shah JP, Patel SG, Ganly I (2017). Management and outcome of clinically evident neck recurrence in patients with papillary thyroid cancer. Clin Endocrinol (Oxf).

[6] Chinn SB, Zafereo ME, Waguespack SG, Edeiken BS, Roberts DB, Clayman GL (2017). Long-term outcomes of lateral neck dissection in patients with recurrent or persistent well-differentiated thyroid cancer. Thyroid.

[7] Brose MS, Nutting CM, Jarzab B (2014). Sorafenib in radioactive iodine-refractory, locally advanced or metastatic differentiated thyroid cancer: a randomised, double-blind, phase 3 trial. Lancet.

[8] Schlumberger M, Tahara M, Wirth LJ (2015). Lenvatinib versus placebo in radioiodine-refractory thyroid cancer. N Engl J Med.

[9] Lim H, Devesa SS, Sosa JA, Check D, Kitahara CM (2017). Trends in thyroid cancer incidence and mortality in the United States, 1974-2013. JAMA.

[10] Dal Maso L, Bosetti C, La Vecchia C, Franceschi S (2009). Risk factors for thyroid cancer: an epidemiological review focused on nutritional factors. Cancer Causes Control.

[11] Kitahara CM, McCullough ML, Franceschi S (2016). Anthropometric factors and thyroid cancer risk by histological subtype: pooled analysis of 22 prospective studies. Thyroid.

[12] Pellegriti G, Frasca F, Regalbuto C, Squatrito S, Vigneri R (2013). Worldwide increasing incidence of thyroid cancer: update on epidemiology and risk factors. J Cancer Epidemiol.

[13] Durante C, Haddy N, Baudin E (2006). Long-term outcome of 444 patients with distant metastases from papillary and follicular thyroid carcinoma: benefits and limits of radioiodine therapy. J Clin Endocrinol Metab.

[14] Hamilton SN, Tran E, Berthelet E, Wu J (2017). The role of external beam radiation therapy in well-differentiated thyroid cancer. Expert Rev Anticancer Ther.

